# Analyzing Immunoglobulin Repertoires

**DOI:** 10.3389/fimmu.2018.00462

**Published:** 2018-03-14

**Authors:** Neha Chaudhary, Duane R. Wesemann

**Affiliations:** ^1^Division of Rheumatology, Department of Medicine, Immunology and Allergy, Brigham and Women’s Hospital, Harvard Medical School, Boston, MA, United States

**Keywords:** B cell repertoire, next-generation sequencing, statistical analysis, immunoglobulin, repertoire

## Abstract

Somatic assembly of T cell receptor and B cell receptor (BCR) genes produces a vast diversity of lymphocyte antigen recognition capacity. The advent of efficient high-throughput sequencing of lymphocyte antigen receptor genes has recently generated unprecedented opportunities for exploration of adaptive immune responses. With these opportunities have come significant challenges in understanding the analysis techniques that most accurately reflect underlying biological phenomena. In this regard, sample preparation and sequence analysis techniques, which have largely been borrowed and adapted from other fields, continue to evolve. Here, we review current methods and challenges of library preparation, sequencing and statistical analysis of lymphocyte receptor repertoire studies. We discuss the general steps in the process of immune repertoire generation including sample preparation, platforms available for sequencing, processing of sequencing data, measurable features of the immune repertoire, and the statistical tools that can be used for analysis and interpretation of the data. Because BCR analysis harbors additional complexities, such as immunoglobulin (Ig) (i.e., antibody) gene somatic hypermutation and class switch recombination, the emphasis of this review is on Ig/BCR sequence analysis.

## Introduction

Analysis and interpretation of antibody repertoire data require an understanding of the complex processes of somatic antigen receptor gene dynamics. Antibodies are composed of a combination of two identical heavy (H) and two identical light (L) immunoglobulin (Ig) chains, each with variable (V) and constant (C) regions. The IgH V-region is encoded by an exon that is generated somatically from assembly of three gene segments, named variable (also abbreviated as V, not to be confused with the V segment-containing V exon), diversity (D), and joining (J) gene segments. The IgH locus contains many related, but distinct V_H_, D_H_, and J_H_ gene segments, which are genomically organized in tandem and selected in a semi-random process for somatic V(D)J assembly in bone marrow progenitor (pro-) B cells. There are two IgL loci—namely, Igκ and Igλ—which have their own pools of tandemly arranged V_L_ and J_L_ gene segments that are assembled by VJ recombination in precursor (pre-) B cells after productive IgH assembly (1, 2). Non-templated (N) and palindromic (P) nucleotides are added to inter-segment junctions, further adding to the diversity. V(D)J recombination is dependent upon Rag1 and Rag2, occurs at the IgH locus before the IgL loci, and Igκ is usually attempted before Igλ assembly. V(D)J recombination usually occurs in an allelically ordered way. In this regard, if a V exon assembly attempt does not result in a productive reading frame, a subsequent attempt occurs on the sister allele. This process results in B cells monoallelically expressing one B cell receptor (BCR) specificity, although rare cells expressing IgH from two alleles, as well as both Igκ and Igλ, have been observed as well (3, 4). Although the IgH and IgL alleles that assemble non-productively do not produce protein, they are transcribed to contribute to the mRNA pool of the cell. Non-productive Ig sequences that appear in sequence data sets can be identified as such in the data processing stage.

Productive assembly of both IgH and IgL chains results in IgM expression on the surface of immature B cells, forming the antigen-binding part of the BCR. Mature naïve B cells express both IgM and IgD due to alternative C_H_ splicing of C_µ_ and C_δ_. Upon activation, B cells can undergo two other forms of diversification, both initiated by activation-induced cytidine deaminase (AID). DNA cleavage and repair events can result in IgH class switch recombination (CSR), where removal of C_H_ region DNA positions alternative C_H_s (e.g., Cγ, Cε, Cα) downstream of the V exon. AID is also required for V exon somatic hypermutation (SHM), which typically occurs in activated germinal center (GC) B cells (5, 6). B cells can further differentiate into BCR-expressing memory B cells, or antibody-secreted plasma cells (7).

**Figure 1:**
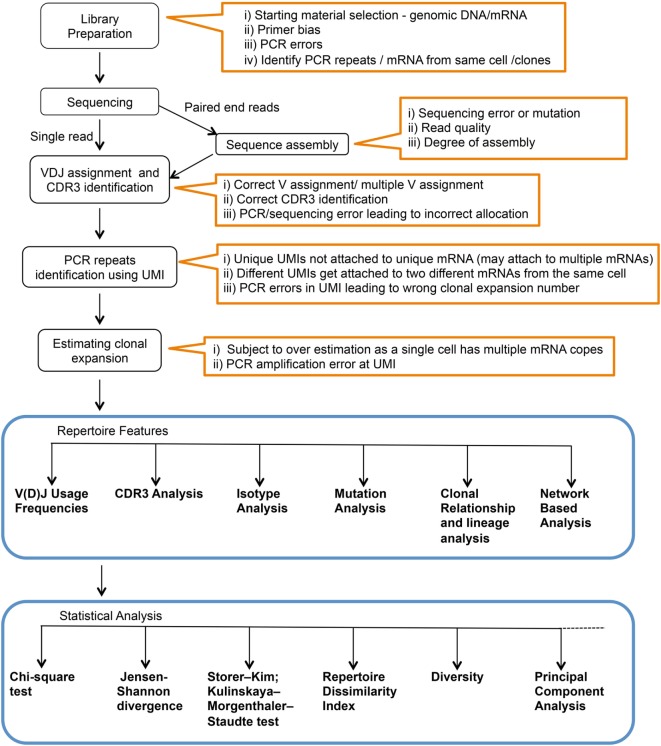
Complete workflow for high-throughput sequencing and analysis of the immunoglobulin repertoire. Text within orange outlines the complications at each step.

While the actual BCR diversity is not completely defined, estimates of the theoretical diversity enabled by V(D)J recombination number more than 10^13^ different potential specificities (8). In addition, only 2% of the BCR repertoire is accessible in circulation at any given time (9). The high diversity and the accessibility limitations constrain our ability to measure and analyze the human immune repertoire. Moreover, what can be learned from deep Ig sequencing is highly dependent upon sample preparation and statistical analysis utilized. In this context, various methods have been described for Ig library preparation and sequencing, and there are numerous statistical tools that have been applied to data analysis (Figure 1). Here, we will briefly review Ig library preparation and sequencing platforms and provide a more in-depth treatment of available analysis tools.

## Library Preparation

Sample library preparation involves the isolation and amplification of the target nucleic acid fragments for sequencing. There are two starting materials that can serve as the initial template to sequence Ig repertoires—genomic DNA (gDNA) and mRNA. Use of gDNA as a template has the advantages of the superior stability of DNA over RNA and the fact that the initial Ig gene copy number is constant between cells. The use of mRNA as an initial template requires an additional step to convert RNA to DNA *via* reverse transcription (RT). Unique Molecular Identifiers (UMIs) can be added to cDNA molecules at this step. UMIs are randomly generated sequences of specific length (usually between 8 and 22 nt) designed to mark individual molecules. These help identify PCR repeats in the analysis, as all repeats from single mRNA will have same UMI. Using mRNA as a template also has the advantage of being intronless, enabling the sequencing of both V and C regions in the same sequence read fragment. Because the number of mRNAs per cell is much higher than DNA copies, the copy number per cell overestimates the number of cellular clones. Despite these disadvantages, the greater mRNA copy number per cell enhances sequence coverage and allows variable and constant region information to be captured on the same length of read (10).

A key objective of techniques designed so far in deep sequencing of Ig repertoires has been to exhaustively amplify the Ig repertoire with minimum error and bias. Primer selection, especially at the 5′ V-region end, is a crucial step to this process as there are many dozens of V gene segments. Some approaches use a mixture of degenerate V_H_ family primers (frame work region 1) as forward primers and a mix of J segment or C region reverse primers. Using a mixture of primers may lead to biases in priming and amplification. Furthermore, SHM-mediated sequence differences may also contribute to unwanted bias (11). The use of synthetic repertoires as control templates to identify and remove potential bias at the analysis stages have been used as an approach to address the problem of primer bias for T cell receptor (TCR) sequencing (12). Another way to reduce primer bias is with the use of 5′ adaptor sequences. This can be done by attaching an oligonucleotide to the 5′ of Ig mRNA molecules by RNA ligation, or by 5′ rapid amplification of cDNA ends (5′ RACE). This enables the attachment of a known sequence to the 5′ end, for use in subsequent PCR amplification steps (13). This approach requires only one set of gene-specific primers targeting the less variable J or C region sequences at the 3′ end. However, 5′ RACE is less able to represent the richness of the sample due to lower efficiency of sequence capture compared to direct priming. The bait capture method uses polyA and part of the sequence of interest attached to streptavidin magnetic beads to isolate the Ig mRNA. The beads are then washed, and the hybridized fragments eluted for sequencing (10). A more recent method called linear amplification-mediated high-throughput genome-wide translocation sequencing (LAM-HTGTS) uses translocation specific sequence at the 3′ end of J region to capture and isolate the complete V(D)J sequence from the gDNA after DNA fragmentation *via* sonication (14). Random fragmentation used with LAM-HTGTS risks losing rare clones. Direct comparison of multiplex PCR, RACE, and bait capture methods for Ig repertoire sequencing showed that these methods were generally concurrent (10).

**Figure 2:**
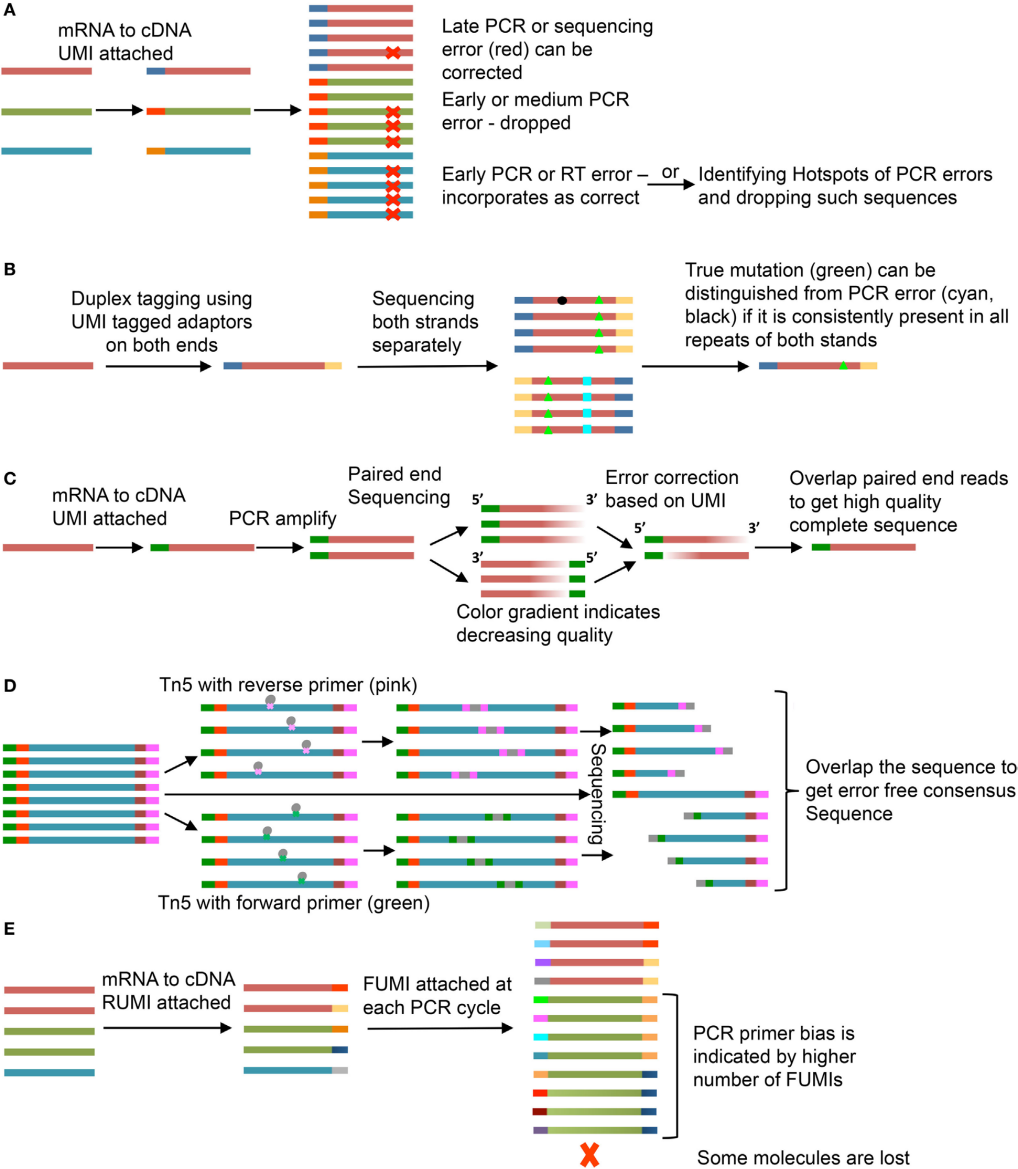
Use of unique molecular identifiers (UMIs). Each strand is an mRNA or a cDNA and smaller bars are UMIs. Same color of the strand and bar represents copies of same mRNA and UMI, respectively. **(A)** Molecular Identifier Group based Error Correction (MIGEC) (17). Among all sequences with same UMI, only few have error (late PCR error) (red), the error is identified and removed; if near 50% of the sequences have the same error, the sequence is dropped; an early error (present in most sequences) would be unidentifiable but it is dropped if it falls on a PCR hotspot. **(B)** Duplex Sequencing (18). UMIs are added to both ends of the sequence and both strands are sequenced. If a mutation (green, black, or cyan) is present in only one of the two stands, it is an error. **(C)** Paired-end sequencing is done after UMI tagging. Error corrections are done for individual reads and then they are merged to get the full good quality sequence (19). **(D)** Tn5-enabled molecular identifier-guided amplicon sequencing (TMIseq) (20). The PCR amplified libraries are tagmented using Tn5 transposase where either forward (green) or reverse (pink) primer is inserted. Thus, only part of the sequence containing both forward and reverse primers gets amplified for sequencing. Both, the smaller libraries and the complete sequence library are sequenced and used to generate a consensus error-free sequence. **(E)** Molecular amplification fingerprinting (MAF) (21). A reverse UMI (RUMI) is added at the reverse transcription (RT) step and a forward UMI (FUMI) is added at each subsequent PCR amplification step. FUMIs keep track of PCR bias for different sequences. Some sequences are over amplified while some may be lost in the process.

Errors may be introduced into the sequence at several steps, including RT, PCR amplification, or during sequencing due to incorrect base call (15, 16). To control for errors that occur during PCR amplification, the UMI can be used to create a consensus sequence of PCR repeats (Figure 2A). A number of UMI-based methods have been devised to improve sequence quality (Figures 2B–D) or identify PCR bias (Figure 2E)—discussed here.

The Molecular Identifier Group based Error Correction (MIGEC) groups similar sequences with same UMI and uses a set of rules to predict errors (17). One rule is to identify a consensus sequence based on the most common variant within a UMI group. However, if the porportions of mismatches are such to evade consensus, the sequence is dropped. A problem with this is that an early error during library preparation could provide a consensus that does not reflect the original template. To solve this, discarded sequences are assessed for PCR error hot spot locations. Sequences with changes within identified error hotspots can then be reevaluated (Figure 2A).

Duplex Sequencing adds UMI to both ends of the sequence and then sequences both strands separately (18). A mismatch has to be present in both the strands to be considered a true mutation (Figure 2B). Another method uses paired-end sequencing wherein both the forward and reverse strands are sequenced after adding a single UMI (19). Errors are removed for both the strands separately and they are overlapped to get the complete sequence (Figure 2C).

Another system uses a sequence target for Tn5 transposase attached to the forward or reverse primer. This allows random insertion into the UMI-containing sequence library (20). The complete sequence and the Tn transposase-foreshortened sequences can be overlapped to get the consensus sequence with less chances of error (Figure 2D). In molecular amplification fingerprinting (MAF), a reverse UMI (RUMI) is added at the RT step and a forward UMI (FUMI) is added with each PCR cycle keeping a track of the number of PCR cycles and PCR bias toward different sequences (Figure 2E) (21). The utility of each of these methods depends on the question under study. The most commonly applied methods of the five are MIGEC and paired-end sequencing. These are the simplest in terms of sequencing and preprocessing steps. If a more stringent analysis of SHM has to be done, Duplex Sequencing and Tn5 transposase method would be expected to offer increased accuracy. In case of MAF, addition of a FUMI at each PCR step would lead to gradual increase in length accompanied by reduced quality at the RUMI sequence site but can be used to understand PCR bias and loss due to random subsampling during sequencing.

**Figure 3:**
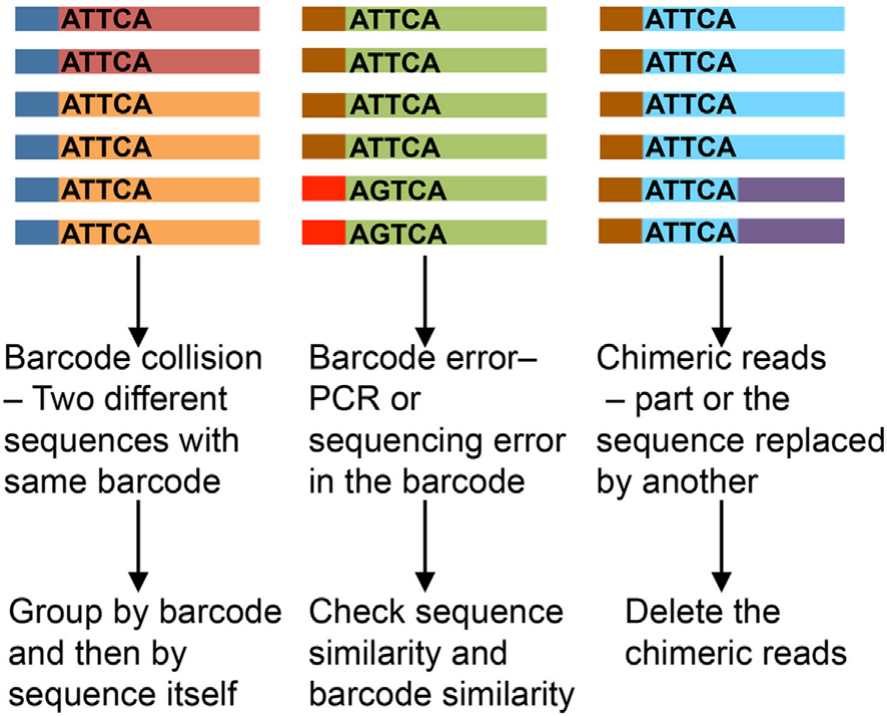
Impact of erroneous barcodes (25). Each strand represents a mRNA. The bar at the end represents a unique molecular identifier (UMI). Same color of the strand and bar represents copies of same mRNA and UMI, respectively. The sequence of the UMI is mentioned within each strand.

Unique molecular identifier length affects the analysis results. Shorter UMIs lead to more non-unique attachment, where the same UMI sequence gets attached to different template molecules. Longer UMIs increase the risk of primer dimer formation and have higher chances of error during amplification and sequencing, which may lead to inflation, misinterpretation, and/or mismatch (22, 23). A UMI length of 8–12 nucleotides is most recommended. Assumptions usually held in the analysis are that UMIs are uniformly represented and all templates uniformly tagged. In practice, however, different target templates have been observed to attach to identical UMI sequences (24). Even with different methods being applied to overcome these issues (23, 25), the impact of erroneous barcodes (Figure 3) may not be trivial (26). We favor an approach of identification of PCR repeats by using both UMI and sequence information (with 1–2 nucleotide error).

**Figure 4:**
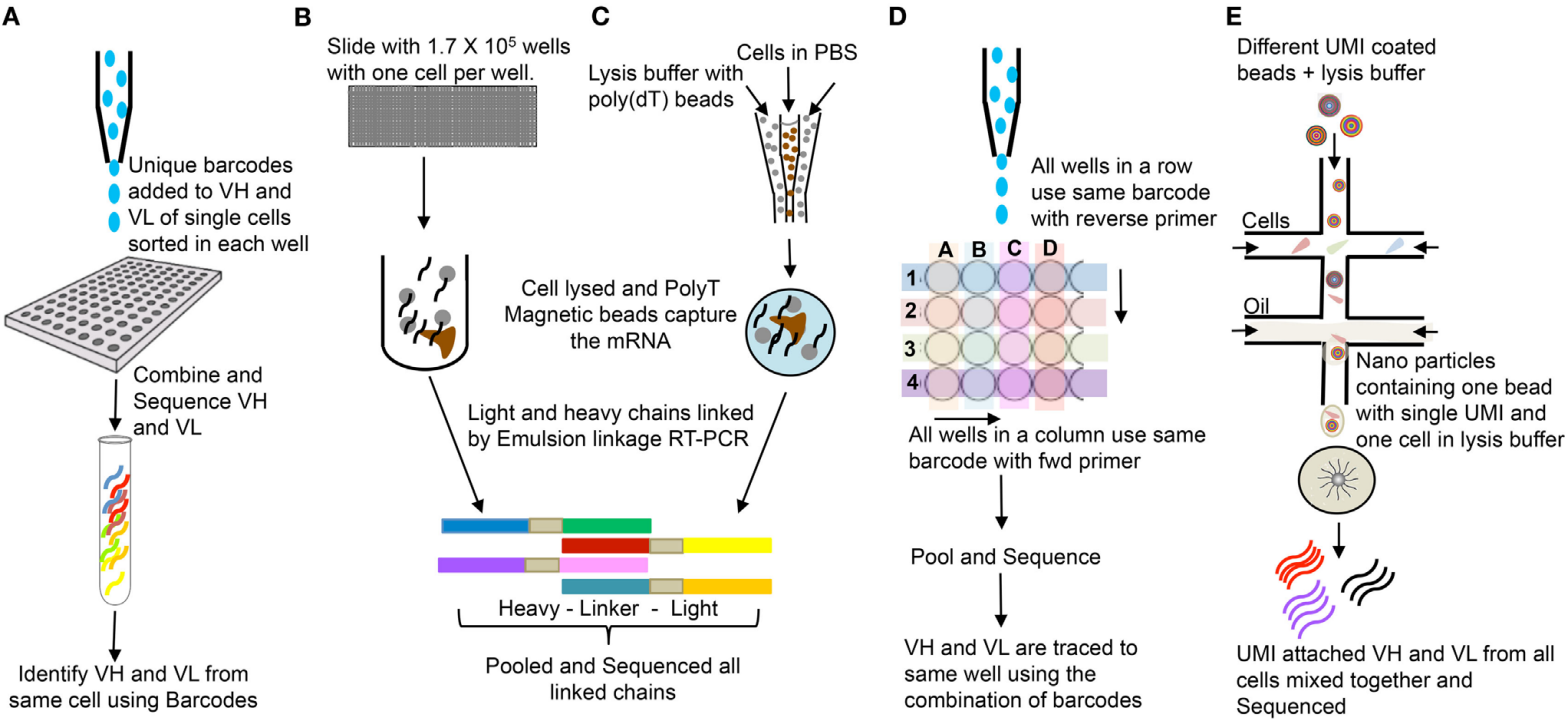
Single cells bulk sequencing: **(A)** Single cells are sorted in 96-well plates, and VH and VL are tagged with cell specific unique molecular identifier (UMI). Sequences from all cells are pooled together and sequenced (29). **(B)** Single cells are isolated in polydimethylsiloxane slides (1.7 × 10^5^ wells/slide-56-μm diameter wells); poly(dT) microbeads are added; wells are sealed with dialysis membrane and equilibrated with lysis buffer; VH and VL mRNAs get attached to poly(dT) beads; beads are emulsified for cDNA synthesis; linkage PCR generates paired VH:VL products which are pooled together and sequenced (30). **(C)** Single cells and poly(dT) magnetic beads are trapped into emulsions along with lysis buffer. VH and VL mRNAs annealed to poly(dT) beads and sequenced as in **(B)** (31). **(D)** Single cells are sorted in 384-well PCR plates. Instead of unique UMI for each cell, each row and column has unique UMIs attached to respective forward and reverse primers, which help trace back to the wells (32). The DNA is pooled and sequenced. **(E)** Microfluidic device joins two aqueous flows into distinct droplets: one with cells and other with barcoded primer beads in lysis buffer. The cell is lysed and its mRNAs hybridizes to the primers on the microparticle surface. The microparticles are collected, washed, and the mRNAs are reverse transcribed, each with unique UMI from the beads. They are pooled and bulk sequenced together (33).

PCR/primer bias for certain templates can complicate assignment of repeat sequences (27). In addition, different B-lineage cell populations can produce widely different amounts of Ig mRNA molecules per cell. In this regard, an activated B cell or plasma cell has a much higher copy number of mRNA than a naïve or memory B cell (28). Assigning identical Ig sequences to clonal expansion versus copies per cell typically requires single-cell sequencing. In addition, IgH and IgL can be paired accurately in single-cell sequencing. A growing number of single-cell sequencing techniques for Ig and TCR repertoire analysis are becoming available. These usually entail an initial barcoding step before amplification and sequencing. Summaries of high-throughput single-cell sequencing approaches are shown in the Figure 4.

## Sequencing Platforms

A number of sequencing platforms are available that differ in features like read length or the coverage of Ig gene, sequencing depth, cost, and run time (Table 1). The PacBio platform, due to its long read length, enables the amplification of H and L chains physically linked together, but is limited due to high error rate, high cost, and low reads per run. Illumina HiSeq offers the highest read depth, but at a cost of read length. Table 1 illustrates the most commonly used platforms along with some of the important features. Larger read number provides higher coverage of a particular sequence giving greater chances of error correction in sequence. Some platforms also provide the feature of paired-end sequencing, in which sequencing is done from both ends of the DNA amplicon, and the final sequence is obtained by merging the two paired-end reads. This ensures superior read quality compared to single end sequencing. Illumina and Ion torrent provide paired-end sequencing. Choice of sequencing platform depends upon the research goals and experimental questions.

**Table 1:** Common platforms used for immunoglobulin repertoire sequencing.

Platforms	Roche’s 454 GS FLX	Illumina MiSeq	Illumina HiSeq	PacBio	Ion torrent
Mechanism	Pyrosequencing	Dye terminator sequencing	Syntdesis (fluoresces attached to nt is excited and detected after each run)	Syntdesis (florescence tag attached to phosphate chain)	Syntdesis (detect H^+^)
Read length	700 bp	300 × 2	250 × 2	860–1,100	>100
Run time	18–20 h	26 h	8 days	0.5–2 h	2 h
Reads/run	1M	3.5M	2B	0.01M	60–80M
Error rate (%)	1	~0.1	~0.1	~13	~1
Type of errors	Indel	Substitution	Substitution	Indel	Indel
Cost/mbp ($)	12.40	0.74	0.10	11–180	<7.5
Region of antibody covered	FWR1-CR	FWR1-CR	FWR1-CR	Amplification of linked H and L chains	FWR3 to CR

## Initial Processing and Annotation

The output for each of these platforms is a binary file format: standard flowgram format (.sff—Roche’s 454 GS FLX), base call (.bcl—illumina), and Binary Alignment Map (BAM—PacBio). Ion torrent gives output in three formats—BAM, FASTQ, or VCF. Each of these has to be converted to Fasta or Fastq format either by running scripts that are part of the software platform (sffinfo-Roche; bcl2fastq-Illumina) or by using one of the many freely available scripts (bamtoFastq, sff_extract). Fasta and Fastq are the two common input formats for most analysis programs. Fasta format consists of a list of sequences with a unique identification tag preceding each sequence. Fastq files (34) also include the information regarding the quality of each residue in the sequence in the form of a Phred score (*Q* score). The *Q* score gives an estimated probability of error for each nucleotide position. They are encoded in the form of ASCII characters, which can be transformed into integers.

Once the data are available from the sequencing reaction, initial processing (often termed “preprocessing”) of the sequences is necessary prior to annotation. Preprocessing includes filtering out low quality sequences, sequence trimming to remove continuous low quality nucleotides, merging paired-end sequences and, if possible, identifying and filtering out PCR repeats. The quality of the output sequences from various platforms is such that with increase in length from the 5′ toward the 3′ end, the quality of residues deteriorates. With Ig sequences, it is important to identify the mutations from sequencing errors. Thus, low quality residues, usually those with a *Q* score <30, at the 3′ end are excluded. In the case of paired-end sequencing, regions of sequence that are included in both reads (i.e., overlapping regions) can be used to form a consensus based on *Q* scores derived from both reads. Sequences with very long stretches of poor quality and paired-end sequences with no overlapping regions are excluded. High-quality filtered and merged sequences can be grouped based on common UMIs (if available from the library preparation), as discussed above, to filter out PCR repeats. In addition, appropriate steps have to be taken to remove sequences with barcode error and remove chimeric reads (25).

Most analysis methods use alignment of the sequence with the germline to assign the respective V, D, and J segments. IMGT database (35) is the most extensively used database for germline Ig sequences. IMGT (36) and IgBlast (37) are the most common annotation software packages, and both use the IMGT database to align sequences. Though alignment with germline seems straightforward, the presence of SHM can make identification problematic as some V gene segments are very homologous and differentiating between allelic differences in the germline and somatically generated mutations may not be straight forward. Also, Ds and Js are small and have insertions and deletions as a result of V(D)J recombination. In many cases, the D segment remains unidentified due to its small size or several can multimerized in tandem (38). Accuracy of gene segment identification depends upon completeness of the reference germline databases. Humans and mice have the most well defined Ig gene loci, but a map of all allelic variants is not complete (39). There have been efforts to address this with algorithms—such as TIgGER (40), IgDiscover (39), IMPre (41), and a more recent allele prediction and validation tool (42)—that can be used to identify germline alleles for individual repertoires. Proper identification of non-template additions and deletions depends to a large degree on the accuracy and completeness of the reference database used.

Apart from IMGT and IgBlast, other software programs are available for analysis of the BCR and TCR repertoire data. A number of them also include preprocessing, annotation, and statistical analyses all in a single pipeline. Some of these programs along with their features are listed in Table 2.

**Table 2:** Softwares available for sequence error correction, annotation, and analysis of immunoglobulin (Ig) repertoire.

Name	Platform/availability	Input format	Maximum sequence limit	Features	Reference
IMGT/V-QUEST	Online	Fasta	50	V(D)J Annotation, junction analysis; mutation; amino acid statistics; comparisons between two repertoires	(36, 43, 44)
IMGT/HighV-Quest	Online	Fasta	150,000		(45–48)

JOINSOLVER	Online/standalone	Fasta	–	Annotation; complimentary determining region 3 (CDR3); mutation; insertion deletion in human only	(49)

VDJSolver	Online	Fasta	500	Use hidden Markov model (HMM) or maximum likelihood to prediction V(D)J recombination	(50)

iHMMune-align	Online/standalone	Fasta		HMM to model the processes involved in human IGH gene rearrangement and maturation	(51)

VDJFasta	Standalone	Fasta	–	HMM-based CDR identification; translation and alignment; probabilistic germline classification	(52)

BASELINe	Online/standalone	Fasta	–	Quantifying selection based on somatic hypermutation (SHM) patterns	(53)

IgAT	Standalone (windows)	IMGT output files	150,000	Gene segments usage; CDR3; antigen selection based on SHM; the hydrophobicity of antigen-binding sites; structural properties of the CDR-H3 loop using Shirai’s H3-rules	(54)

IgBlast	Online/standalone	Fasta	Online-1,000/SA-none	V(D)J assignment; CDR3 identification; mutation; can use custom database in SA	(37)

pRESTO	Standalone	Fastq/Fasta	None	Merge; filter; error correction (with/without UMIs); annotation	(55)

Vidjil	Online/standalone	Fastq/Fasta	None	Extract V(D)J junctions; clonality	(56, 57)

The antibody mining toolbox	Standalone	Fastq	None	Analysis based on CDR3 as sequence identifiers	(58)

MIGEC	Standalone (Unix)	Fastq	None	Error correction and sequence assembly	(17)

IgRepertoireConstructor	Standalone	Fastq	None	Merge; filter; error correction (with/without UMIs); validation using mass spec; clonality; diversity	(59)

MiXCR	Standalone	Fastq	None	Merge; filter; PCR error correction; annotation; Gene segment usage; clonality; mutation	(60)

IMonitor	Standalone	Fastq/Fasta	None	Merge; filter; V(D)J assignment; gene usage frequency; CDR3; mutation; insertion and deletion	(61)

IgSCUEAL	Standalone	Fasta	None	V J annotation based on phylogeny; gene usage frequency; CDR3 length	(62)

Change-O	Standalone	IMGT/IgBlast Result	None	Gene usage; clonality; CDR3; diversity; phylogenetic; mutation; selection pressure; novel germline prediction	(63)

TIgGER	Standalone	Fasta	–	Predicts germline alleles	(40)

LymAnalyzer	Standalone	Fastq	None	V(D)J identification; CDR3; diversity; mutation; polymorphism analysis	(64)

sciReptor	Standalone	SFF/Fastq/Fasta	2,500	Single-cell analysis, annotation; maintains regional database; gene segment usage; clustering; mutation	(65)

repgenHMM	Standalone	Fasta	None	Predicts scenarios of V(D)J recombination	(66)

bcRep	Standalone (R)	IMGT output files	–	Gene usage frequency; clonality; diversity; mutations; repertoire comparison; visualization	(67)

IgDiscover	Standalone	Fastq	–	Identification of existing and novel germline V genes	(39)

Recon	Standalone	Frequency table (txt)	–	Diversity	(68)

IMPre	Standalone	Fasta	–	Predicts germline genes and alleles	(41)

ARResT/Interrogate	Standalone	IMGT output files	–	Calculation of statistics; visualization	(69)

Antigen Receptor Galaxy	Online	Fastq/Fasta	None	Demultiplex; annotation using IMGT/High V-Quest; V(D)J usage; SHM and CSR; Ag selection; clonality	(70)

IGoR	Standalone	Fasta	None	Calculates V(D)J recombination and mutation probabilities	(71)

ClonoCalc and ClonoPlot	Standalone	Fastq	–	GUI; Demultiplex; merge and annotate using MiXCR; analysis and plots using tcR package in R	(72)

## Describable Features of B Cell Repertoires

The expansive capacity of the BCR repertoire makes the probability of finding the same sequence within two individuals and even within two tissues of same organism extremely low, and this limits direct comparisons of specific sequences between individuals. However, it has recently been shown that human TCR repertoires can be grouped into functionally related categories that can be shared between individuals (73). The same algorithm, called GLIPH (Grouping of Lymphocyte Interactions by Paratope Hotspots), could also be used to group functional BCR repertoire but would have to include the additional complexity due to SHM. A number of other features have been used to quantitatively compare antigen receptor repertoires between individuals, groups, or experimental conditions. Below, we provide a brief survey of measurable repertoire features and some representative studies that have assessed them in the context of a variety of lines of inquiry.

### V(D)J Segment Usage Frequencies

An Ig repertoire can be described in terms of the frequencies with which it uses the gene segments that make up the V exon, particularly the V segment, as it is the longest and most diverse. V gene segment frequencies, or VJ combinations frequencies, have been used to compare stages of immune responses, for example, to describe differences in B cell repertoires of avian flu (H7N9) patients at the time of infection and during recovery, where recovery was shown to utilize more diverse VJ combination frequencies (74). V gene usage frequency comparisons have also been used to describe age-related changes (75) as well as general population level descriptions (39, 40, 76).

### Complimentary Determining Region 3 (CDR3) Properties

The CDR3 is the most variable region of an antibody and can be used to define clonal lineages. The CDR3 length and amino acid properties have been used to characterize a functional repertoire. The advantages and methods of CDR3 comparisons are reviewed elsewhere (77). There are many studies comparing CDR3 features in repertoire analysis. Comparisons of CDR3 lengths between cell groups expressing different IgH isotypes showed that IgM had longer CDR3s compared to all other isotypes examined (11), suggesting a potentially interesting link between a general V-region feature and IgH isotype. An analysis of BCR repertoire of naïve, IgM memory, and class switched memory B cells suggest that memory B cells may have shorter CDR3s with more positively charged amino acids. It was also found that IgM memory cells may have lower hydrophobic and aliphatic indexes compared to memory cells of other IgH isotypes (78). Antigen-experienced B cell repertoires appear to have a more exposed CDR3 region rich in charge (79). Antigen exposure also appears to be associated with a decrease in CDR3 length (80). IgM and IgA CDR3s tend to be longer with age (81). Systemic lupus erythematosus (SLE) patients were reported to have shorter CDR3 with higher arginine content (82).

Complimentary determining region 3 analysis also helped identify the “public” sequences. Public CDR3 (or public Ig) is a term used when similar or identical sequences are found in different individuals. They are usually reported in individuals who had been exposed to the same pathogen, like *Haemophilus influenzae* type B, tetanus toxoid, and influenza (83, 84). Public sequences are more common for IgL as compared to IgH (79). The public BCRs have also been observed in persistent diseases like autoimmunity and cancer (85). Understanding emergence of public CDR3s could help understand the process of affinity maturation and antibody development (86).

### Mutation Analysis

Diversity due to somatic mutation is also a feature of the Ig repertoire. This includes insertions and deletions during V(D)J recombination and SHM. During SHM, AID targets at DGYW motifs (*D* = A/G/T, *Y* = C/T, *W* = A/T) (87, 88), which are also referred to as mutational hotspots. In general, mutations are analyzed as degree of divergence from germline sequences and give insight into the biological process of SHM and affinity maturation. Any nucleotide mutation can result in a different amino acid encoded at that position (replacement) or can result in no change (silent). Analysis of replacement versus silent mutation status at nucleotide positions can have implications for studies examining positions important for antibody selection (53, 89).

Somatic hypermutation analysis in twins has shown that genetic factors play a role in determining mutation frequency (90). Similar analysis showed that the level of SHM is reduced in older individuals (81). AID-mediated mutations tend to occur unequally across the V exon. CDRs have more hotspots and tend to mutate more than FWRs. Also, mutation selection pressure is different for the two regions. Mutations in the FWRs are more likely to be selected against, as these regions are important for structural fitness (91). Insertions and deletions occur during SHM, adding to the structural plasticity of the antibodies, but are relatively rarely found as they are more likely than mutations to cause negative selection from structural instability (92).

Somatic hypermutation studies have been employed to decipher why Ig loci are permissive for AID-mediated mutation compared to off target, non-Ig loci. This remains one of the most elusive questions in B cell biology. Studies examining a particular V gene segment in which certain AID-target hot spots were experimentally removed in a mutating human B cell line suggested that local sequence context may influence SHM of other regions within the V exon (93). Local sequence context was also shown to influence AID targeting on a passenger allele system, wherein a non-productive test allele was paired with a productive IgH knock-in to remove the effects of BCR-mediated cellular selection (88). DGYW motifs within CDR sequence regions were in general targeted more than DGYW motifs in framework regions (88). When the Ig passenger sequence was replaced with a non-Ig sequence, it was also targeted by AID, suggesting that the general location of the Ig V-region in the context of the IgH locus was an important feature of accessibility to SHM (88). This same passenger allele system was used to uncover sequence-intrinsic SHM-targeting rates of nucleotides across substrates representing maturation stages of an anti-HIV-1 broadly neutralizing antibody (94).

### Isotype Analysis

Immunoglobulin repertoire analysis can provide insights into the biology of IgH isotypes. Each isotype has distinct biological functions governed by the C_H_ region domain. The sequences in a repertoire can be categorized into their respective isotypes if the experimental design accommodated for C region sequence in the library. Isotype analysis has included the categorization of Ig repertoire features, functions, or conditions to Isotype groups. As discussed above, sequencing data have shown that IgM is the least mutated and features the longest CDR3 in general compared to the other isotypes (11). Among memory cells, IgM has lower hydrophobic and aliphatic index compared to others (78), and SHM frequency has been reported to be higher in switched isotypes compared to IgM and IgD and varies between different subclasses of the same isotype (11). Isotype and SHM analysis has also been a key part of the concept of sequential switching. C_H_ regions for the various IgH isotypes are arranged in tandem along the IgH locus. Sequential switching occurs when CSR occurs first to Cμ-proximal C_H_ regions (e.g., to produce IgG3, IgG1, or IgA1), and then from these, to distally located isotypes (e.g., to IgG2, IgG4, or IgA2) (95). Studies have indicated that direct and indirect CSR can occur to distal isotypes (96, 97).

### Clonal Relationship and Lineage Analysis

Lineage analysis and identification of clonal relationships between antibodies collected from an infected individual or during course of infection over time can track the evolutionary steps in the development of functional antibodies. This has been used in following HIV-1 bnAb VRC01 producing lineage for 15 years using peripheral B cell sampling for the rate of maturation and diversification in a single HIV-1-infected patient (98). A high substitution rate of 2 per 100 nucleotides per year resulted in extreme diversification in the context of chronic infection. Another study involving HIV-1 bnAbs found the intermediate antibodies to have reduced autoreactivity (99). PGT121-134 (100), PGT135-137 (16), and CH103 (101) are other bnAbs against HIV whose lineages have been studied in detail. Ig lineage and clone analysis has shown to have clinical relevance in the setting of lymphoma diagnostics. In this regard, lineage analysis at the time of diagnosis and relapse has revealed that B cells that reemerge are generally clonally related to the original cancer causing BCR (102, 103).

### Network Based Analysis

**Figure 5:**
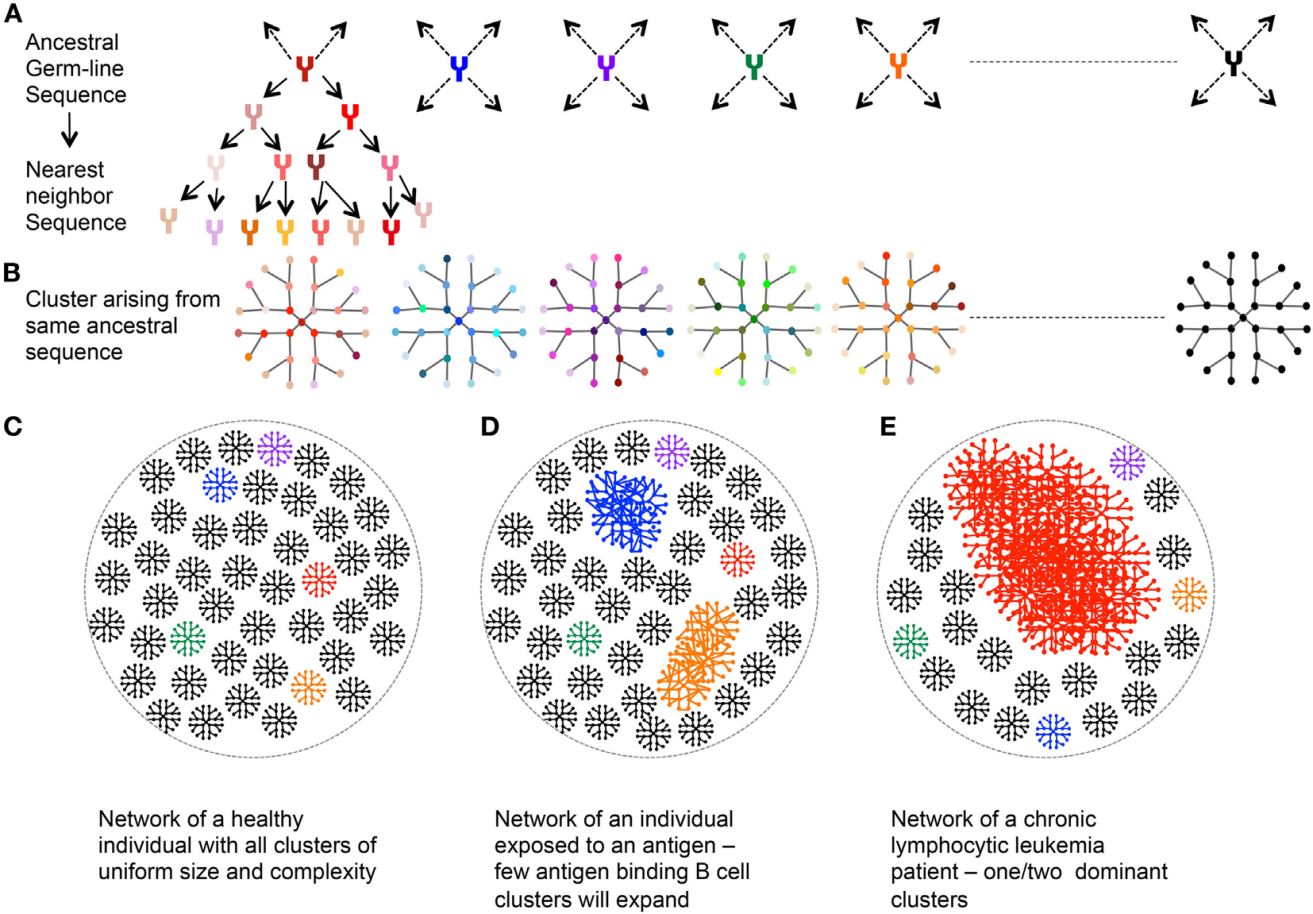
Network analysis of immunoglobulin (Ig) repertoire—an explanatory model. **(A)** An example network arising from single germline sequence (Red). **(B)** Multiple clusters arising from different ancestral sequences. Each color represents cluster arising from different germline. **(C)** Representative network of a healthy individual: each cluster arising from an ancestral sequence is of uniform size and complexity. **(D)** Representative network of an individual exposed to an antigen: larger clusters represent the antibody, which recognizes the antigen and hence expands and mutates. **(E)** Representative Ig network of chronic lymphocytic leukemia patient with one dominant highly expanded cluster.

A network is made from a group of entities (or nodes) connected to each other by links or edges if they share selected features. A B cell network may be based on mature antibody sequences clustered around the germline ancestor sequence. In this regard, all the nodes in a cluster would be the sequences identified to have come from that ancestor sequence, with edges connecting the nearest previous ancestor (Figures 5A,B). A healthy individual should have a very uniform network with each cluster of similar size and complexity (Figure 5C). An individual recently infected with a pathogen would have few expanded clusters corresponding to various versions of pathogen-reactive clones (Figure 5D). A uniformly distributed network versus a deformed network with few overly expanded V_H_ segments can identify chronic lymphocytic leukemia patients (104) (Figure 5E). A simpler network would be based on just the CDR3 region wherein homologous CDR3s are clustered together. Hepatitis B-infected patients harbor specific CDR3 sequences that may serve as identification signatures (105). General network properties—including reproducibility, robustness, and redundancy, have been studied for healthy Ig networks and can be evaluated *vis-a-vis* diseased Ig networks (106). The iGraph package in R can be used for network construction and visualization (107).

### Paired Heavy and Light-Chain Analysis

Single-cell high-throughput sequencing of IgH and IgL together has been an important advance. With knowledge of IgH/IgL pairing, frequencies of paired usage of different V_H_ and V_L_ gene families can be determined together and a more authentic evaluation of antibody specificity can be achieved—as has been done in the evaluation of vaccine responses (30, 108, 109) as well as in autoimmune and inflammatory diseases (110). A comparison of single-cell sequences from naïve and antigen-experienced Ig repertoires uncovered several features related to how IgH and IgL pair together between these two groups (79). Single-cell sequencing can easily identify allelic inclusions, specifically noted by presence of both kappa and lambda light chains on the same B cell, as well as public V_H_ and V_L_ sequences. Single-cell sequencing has also shown that public V_L_s were able to pair with multiple V_H_ in multiple donors (31).

## Statistical Analysis of B Cell Repertoires

Various forms of statistical tools have been applied on BCR and TCR sequences in a descriptive sense as well as to compare them in the context of experimental systems. Some or all of these methods can be used to describe and compare most of the BCR repertoire features discussed above. Below, we provide a brief survey of some of the analysis tools used in Ig repertoire studies.

### Resampling

Resampling otherwise known as rarefication, or subset analysis, is a technique used to correct for differences in sequencing depth between samples. The sequencing reaction may generate more reads in certain libraries due to stochastic reasons and, depending on how sequences are processed, has the potential to generate erroneous conclusions in the analysis. Subset sampling has been used in metagenomics studies where the number of sequences for all samples is reduced to the depth of that with the lowest read count. This step is designed to exclude any differences in the analysis that may be due to variable read depth, instead of the underlying biologic principle under investigation (84, 111, 112). However there are different views regarding use of rarefication. On one hand, subset analysis resolves randomly generated differences in sequence depth, but also results in discarding data, which leads to loss of assay power. This reduces the ability of finding difference between populations. In this light, it is important to run several control subsampling analyses to examine the degree to which the test subsamples reflect the properties of the whole. A sufficiently subsampled library from a whole library of sufficient depth should be essentially identical to the whole as well as other test subsets. Parallel comparisons of subsetted and whole data may be valuable to uncover read depth sufficiency. In general, we use subsetting when comparing averages of feature measurements from experiments repeated independently. If a test is used that considers only total counts (instead of averages of multiple experiments), such as the chi-square test, then we do not subset, as long as control comparisons of independently repeated tests indicate sufficient read depth of individual samples.

### Chi-Square Test

Chi-square test for independence (113) checks if the proportions of two categorical variables are different from each other or not. It is a non-parametric test, which deals only with total counts—relative frequencies are not allowed. Here, the null hypothesis (H_0_) states that the variables are independent while the alternate hypothesis states that they are dependent, i.e., knowledge of one variable can help predict the other variable. The test statistics for the Chi-square test is calculated as:

$$\chi^{2}=\sum \frac{{\left(O-E\right)}^{2}}{E},$$

where *O* is the observed frequencies; *E* is the expected frequencies, which, for each observation in the table is calculated as [(total observations in respective row)*(total observations in respect column)]/total number of observations in the table.

A limitation of the chi-square test is that it is extremely sensitive to sample size. The number of samples has to be large enough to have an expected value of at least 5 in each cell (113). Also, the test becomes more and more sensitive with increase in the sample size—eventually showing significance even with mild variation that can occur within assay error or repeat biological samples (114). This limits the use of chi-square test in high read output platforms, such as the illumina systems. An example of deep sequencing data analyzed with chi-square test is in the comparison of V_H_ and V_L_ segment usage in developing B cells within weanling mouse bone marrow versus intestine. Chi-squared tests of pyrosequencing data showed significant differences in the V_L_ segment usage for the two groups, but not when randomly compared between biological repeats (115). Another such study used the chi-square test on Sanger sequencing data to compare the usage of V, D, and J segment families among patients with chronically evolving hepatitits C Virus (HCV) infection compared to resolved HCV, and healthy controls (116). They found that some of the families showed statistically significant association with the clinical groups for each of the three segments. HIV-1 specific and non-HIV-1 specific antibodies from an infected individual showed differences in the highly used V_H_ segments (117). A comparison of productive and non-productive antibody sequences revealed strong bias in the pairing of specific D and J segments due to multiple sequential D-to-J rearrangements (118). The function for calculation of chi-square statistics is available in the R package “stats.”

### Jensen–Shannon Divergence (JSD)

Jensen–Shannon divergence gives a measure of similarity between two probability distributions (119), and has also been used in Ig repertoire analysis. JSD is derived from Kullback–Leibler Divergence (KLD). For two probability distributions *A* and *B*, the JSD is calculated as:

JSD(A||B)=0.5 *(KLD(A||M)+KLD(B||M)),

where *M* = 0.5(*A* + *B*)–midpoint of the two probability distributions and KLD(*A*||*M*) and KLD(*B*||*M*) are the KLD of *A* and *M*, and *B* and *M*, respectively. JSD is symmetric [JSD(*A*||*B*) = JSD(*B*||*A*)] and non-negative measure in contrast to KLD which is asymmetric [KLD(*A*||*B*) ≠ KLD(*B*||*A*)] and may be negative. JSD = 0, if *A* = *B*. JSD is also a non-parametric test. Since the test compares probability distributions of two populations, it is not affected by sample size. However, the effect of difference in sequencing depths leading to the differences in the probability distributions would still interfere with the results. Unlike KLD, it is symmetric, with values bound between 0 and 1 for both directions of comparison, which simplifies comparisons of multiple distributions.

Some studies, which included the use of JSD, calculated the distance between the repertoires under different conditions. JSD was used to compare TCR repertoires of cells with different epitope specificities (120). Ten epitope-specific TCR repertoires were characterized, and the JSD was used to compare gene frequency distributions for these repertoires with respect to the background distribution. A comparison of VJ combination and VJ-independent repertoires of peripheral blood mononuclear cells (PBMC) and tumor-infiltrating lymphocytes (TIL) in glioma patients revealed specific signature TCRs that were associated with PBMC of patients exhibiting low TIL divergence and which were depleted in patients with highly divergent TIL repertoires. This divergence, detectable in PBMC, can be used as a noninvasive technique for longitudinal monitoring of glioma (121). JSD has also been used to find similarity in isotype abundance in repertoires of individuals (95). The R package “tcR” includes a function to calculate the JS divergence for TCR and BCR repertoires (122).

### Storer–Kim (SK) and Kulinskaya–Morgenthaler–Staudte (KMS)

Storer–Kim and KMS tests have been used recently to find statistically significant differences between two distributions (123, 124). Both tests assume non-parametric distribution. The second assumption might not be appropriate when considering affinity maturation and clonal expansion. SK test does not provide a confidence interval while KMS test does. Like JSD, these tests compare probability distributions and hence there is no limitation to number of sequences. A mouse study used SK and KMS tests to compare the V family usage within GC B cell repertoire of animals vaccinated with complex Ebola virus-like particle and unvaccinated controls (125). Enhanced use of IGHV8 was observed in the vaccinated group. The tests have been implemented in “WRS2” R package (126).

### Repertoire Dissimilarity Index (RDI)

Repertoire dissimilarity index compares Ig repertoire based on usage of V, D, or J (127, 128). It is a non-parametric method, which tries to circumvent the problem of varying number of sequences in different samples. The first step involves subsampling the larger sample to the size of the smaller one. From these uniform samples, the feature of interest is counted; the frequency is normalized and transformed into probability distributions. Root mean square deviation (RMSD) is calculated between the two. Random subsampling is done multiple times and mean RMSD is calculated to get the RDI. This reduces sampling bias effects of rarefication to some degree. Since, for each comparison, the sample size is the lower of the two, RDI values between different samples are not comparable. Also, with decrease in sample size, RDI values increase. The RDI value gets closer to the true value as sample size increases. RDI was used to show that genetic bias effects VJ usage by analyzing BCR repertoire of monozygotic twins (127). RDI was validated by recapitulating known differences between T-cell subsets (128). R codes for calculation of RDI are available at https://bitbucket.org/cbolen1/rdicore (128).

### Diversity

Diversity has frequently been used to describe lymphocyte antigen receptor repertoires. These indices come from ecology, where they are used to compare the diversities of ecosystems. With respect to the immune repertoire, diversity can be calculated in terms of use of V, D, and J gene segments as well as the use of individual CDR3s. Depending upon the kind of comparison diversity can be categorized into three types, namely, alpha, beta, and gamma. Alpha diversity is the diversity of an individual’s repertoire, i.e., the total number of individual species (V_H_ or CDR3) present in the repertoire. This is also the species richness. Beta diversity gives a difference in repertoire of two individuals. It would be given by the sum of unique species in both the repertoires. Gamma diversity is a combine diversity of all the ecosystems or repertoires. Alpha, beta, and gamma diversities were compared between patients with gastritis with (GHP) and without *Helicobacter pylori* (GNHP) background, gastric mucosa-associated lymphoid tissue lymphoma (MALT-L) (caused by GHP), and diffuse large B cell lymphoma (DLBCL) (may or may not be transformed MALT-L) (129). Contrary to the expectation, similar diversification was found in both GHP and GNHP, and MALT-L transformed DLBCL, and independent DLBCL. Also, MALT-L transformed DLBCL and MALT-L patients did not share any feature in their repertoires.

### Species Richness

Species richness (alpha diversity) is the total number of unique species in a community. It is just a count and does not take into account the species abundance. It is the simplest way of describing diversity but is very sensitive to sampling depth. Greater sampling depth results in capture of more and more rare species resulting in higher species richness. Rarification can have a significant impact on this measure, as less represented species are usually lost during random subsampling. To account for the unseen species problem for under-sampled population, a number of measures have been devised which predict the actual species richness based on the sampled data, including Chao1 (130), abundance-based coverage estimators (ACE) (131), and DivE (132).

### Chao1 and Abundance-based Coverage Estimators (ACE)

Chao1 and ACE have commonly been used in assessment of microbial species richness. These estimators add a correction factor to the number of observed species to account for the hidden/unsampled once (133). Chao1, for example, extrapolates the richness based on the number of rare species (count = 1 or 2) found in the samples.

$$\mathrm{Chao1}=S_{\mathrm{obs}}+\frac{{n_{1}}^{2}}{2n_{2}},$$

where *S*_obs_ is the observed number of species, *n*_1_ is the number of singletons (species with count = 1), and *n*_2_ is the number of doubletons (species with count = 2).

Abundance-based coverage estimator, on the other hand, takes into account the number of species with count less than or equal to 10. It is calculated as:

$$\mathrm{ACE}=S_{\mathrm{abund}}+\left(\frac{S_{\mathrm{rare}}}{C_{\mathrm{ACE}}}\right)+\left(\frac{F_{1}}{C_{\mathrm{ACE}}}\right)\gamma_{\mathrm{ACE},}^{2}$$

where *S*_abund_ is the number of species with count greater than 10; *S*_rare_ is the number of species with count less than or equal to 10; *C*_ACE_ = 1 − *F*_1_/*N*_rare_; *F*_1_ is the number of species with count = 1

$$N_{\mathrm{rare}}=\sum_{i=1}^{10}iF_{i};\;F_{i}\text{ is the number of species with count}=i,$$

γACE2=max[Srare∑i=110i(i−1)FiCACE(Nrare)(Nrare−1)−1,0];Coefficient of variations of Fi's.

Even with the correction factors incorporated to calculate the true species richness, these estimators are still sensitive to sampling depth. A small change in the library preparation steps leading to increased sample quality or quantity may impact species diversity measurements. These factors are still unable to predict the real number of unseen species.

### Diversity Estimator (DivE)

DivE (Diversity Estimator) is a diversity measure used originally in the calculation of TCR repertoire diversity (132). The initial step involves construction of rarefaction curves for multiple nested subsamples. A rarefaction curve is a plot of the number of species as a function of the number of sequences or sample size. A mathematical model, defining each of the rarefaction curves, is built and tested on all the nested samples. Each model is scored based on degree of fit using four criteria: Discrepancy (between the data points and the model), accuracy (of predicted versus actual species richness), similarity (between area between the curve fitted to the subsample and the complete data), and plausibility (the predicted number of species should increase or plateau off or the rate of increase of species should decrease or remain constant—any other scenario is not plausible). The top five scored models are extrapolated and combined to calculate a DivE. This estimator is unaffected by sample size and its accuracy is improved from the use of multiple models to predict diversity. The drawback is that the calculation process is lengthy and there is a requirement to fit multiple models. DivE has been used to calculate the species richness of T cell repertoires. With B cell repertoires being even more diverse, the computations are expected to be more complex. This species richness estimator was used to calculate the number of cells infected with human T-lymphotropic virus type 1 in patients, species richness in a TCR repertoire and fecal microbiota of infants (132).

These estimators have been adopted in analysis of diversity of BCR and TCR repertoires. Studies on the effect of aging on the B cell immune repertoire diversity on administration of influenza vaccine showed that the repertoires become more specialized and less plastic with age (134). Both naïve and antigen-experienced repertoires show reduced diversity with age. The Chao1 estimator was used to describe BCR repertoire differences within and between individuals (84). The R packages for estimation of DivE (132), Chao1, and ACE (135) are available.

Although species richness may be the most direct measure of diversity, evenness or the homogeneity/uniformity of species in the community also provides important information. Species evenness would describe the degree of clonal expansion in an immune repertoire. Two common indices calculated considering both richness and evenness, namely, the Shannon Index and the Simpson Indexes have different prospective for each (136).

### Shannon Index (*H*)

Shannon index (*H*) calculations operate under the assumptions that individuals are randomly sampled from an infinitely large community, and that all species are represented in the sample. The Shannon index increases as both richness and evenness of the community increase. The Shannon index is given by:

$$H=-\sum_{i=1}^{s}p_{i}\text{ ln }p_{i},$$

where *p_i_* = *n_i_*/*N* the proportion of individuals of the *i*th species; *n_i_* is the number of individuals of the *i*th species; and *N* is the total number of individuals and s is the total number of species. Since this index is directly proportional to the species richness, it is sensitive to sampling depth.

### Simpson Index of Diversity

Simpson Index of diversity is calculated as 1 − Dominance Index (*D*). *D* gives more weight to dominant species. It gives the probability that two individuals drawn from a population will belong to the same species. Thus, presence of rare species would not affect *D* and *D* increases with increase in dominance leading to decease in diversity. Simpson index of diversity (1 − *D*) gives the diversity value, which increases with decrease in dominance.

$$1-D=1-\frac{\sum_{i=1}^{s}n_{i}\left(n_{i}-1\right)}{N\left(N-1\right)},$$

where *n_i_* is the number of individuals of the *i*th species and *N* is the total number of individuals, and *s* is the total number of species.

Shannon diversity has been used widely in antigen receptor diversity analysis. Some examples of this analysis in human studies include the comparison of TCR repertoires in colorectal tumors and adjacent healthy mucosa (137) and B cell repertoire of patients before and after hematopoietic stem cell transplantation (138). R packages are available for calculation of diversity indices like vegan (139) and BiodiversityR (140), with one developed specifically to characterize and analyze immune repertoires (122). Recon is another program developed to calculate the diversity measures (68).

### Diversity 50 (D50)

Diversity 50 or D50 is the percentage of dominant unique species, which make up 50% of the total community. In terms of Ig repertoire, it is the percentage of distinct V_H_ segments or CDR3 constituting half of the total V_H_ or CDR3 in a population (141). A larger D50 value shows larger diversity. D50, like the Simpson index, is based on the number of dominant species and is not affected by the addition of rare species. The D50 has been used to compare the degree of clonal expansion/clonal dominance during infection. Both T and B cell repertoire diversity have been assessed *via* D50 analysis in human studies of viral infection (74), as well as in the characterization of TCR diversity in patients with Wiskott–Aldrich syndrome (142).

### UniFrac Distance Matrix

In the context of microbial communities, this index includes environmental differences by taking into consideration phylogenetic information (143, 144). The branch lengths are deemed to differ based on genetic changes occurring due to environmental selection pressure. Thus, the branch lengths between two species in both communities are taken into account while considering the distance between the communities. Analogically, different selection pressure within repertoires of two organisms can be taken into account by including the phylogenetic information starting from the germline sequence (134). UniFrac distance is also sensitive to sequencing depth. Smaller number of sequences in a sample would be underrepresenting the rare species and this would artificially influence the distance between similar communities. UniFrac distance was used to calculate the difference between the Ig repertoires before and after immunization with influenza vaccine in old and young individuals. With age, Ig repertoires appear to become more specialized and less plastic, resulting in lower uniFrac distances, compared to younger individuals (134). R packages for calculation of uniFrac distance are available (145, 146).

### Principal Component Analysis (PCA)

Principal component analysis is a way of simplifying the analysis of large datasets by reducing the dataset dimensionality. It does so by creating a new set of variables or principal components (PCs), which describe more complex variability in the data set. The first PC (PC1) explains the maximum variance of the dataset, followed by PC2, and so on. PCA can also help identify patterns in the data, which would otherwise not be prominent. PCA can be used to compare the Ig repertoire based on multiple variables. Using multiple variables like diversity, mutation rates, and others, to define Ig repertoire under different conditions, PCA has been used to find association patterns between these groups. A limitation of PCA is that it considers only linearly correlated data. Also, it discards smaller variance as noise, which may be important under certain conditions. Depending upon the variables being used to analyze the samples, PCA may or may not be dependent on sequencing depth. For example, having diversity as one of the variables would make PCA sample size dependent. PCA has been used on V(D)J usage among productive antibodies to explore the relationship between pre-B, FO, and MZ cells. A very clear clustering and gradient separation of pre-B, follicular, and marginal zone cell subsets was seen which was also observed with V usage analysis but not that of D and J (147). In a study comparing the effect of various influenza vaccines on B cell repertoire, PCA was applied to rarefaction analysis, diversity, V usage frequencies, and mutation rates for unimmunized and immunized groups (148). The basic stats package of R has function for PCA.

When it comes to the analysis of Ig repertoires, a standard protocol has yet to be set. The specific scientific question and the difference in the sequencing depth is one of the major concerns when selecting a statistical approach. Rarefication, a way to overcome differences in sequencing depth, works best when the number of sequences is not very different for each sample. This criterion is not always met. Chi-square test does not work well with sequencing depth of over a few thousand. The JSD, SK, and KMs approaches work are reasonable measures for large sequencing data sets. RDI addresses the problem of variable sequencing read depths by resampling multiple times and taking the mean. However, the RDI values for two different pairs of data are not comparable. JSD on the other hand always gives a bounded value between 0 and 1 and can be relatively scaled between different comparisons. Diversity, though being the most common method used to assess and compare the Ig repertoire, is very susceptible to sequencing depth. Because each estimator used alone incompletely describes the diversity of a B cell repertoire, multiple parallel approaches are warranted.

## Concluding Remarks

High-throughput sequencing provided immunology with a tool to enhance our understanding of lymphocyte antigen receptor repertoires. With increased application in human diagnostics—sample preparation, sequencing, and analysis techniques will continue to evolve to assist workers in describing lymphocyte antigen receptor repertoires. As large data sets become less expensive and more efficiently produced, necessities for more uniform and improved analysis methods are expected to drive further innovation.

## Author Contributions

All authors listed have made a substantial, direct, and intellectual contribution to the work and approved it for publication.

## Conflict of Interest Statement

The authors declare that the research was conducted in the absence of any commercial or financial relationships that could be construed as a potential conflict of interest. The reviewer TT declared a shared affiliation, though no other collaboration, with the authors to the handling editor.

## Abbreviations

Ig, immunoglobulin; BCR, B cell receptor; TCR, T cell receptor; AID, Activation-induced cytidine deaminase; CSR, class switch recombination; SHM, somatic hypermutation; GC, germinal center; UMIs, unique molecular identifiers; JSD, Jensen–Shannon divergence; KLD, Kullback–Leibler divergence; SK, Storer–Kim (KMS); KMS, Kulinskaya–Morgenthaler–Staudte; RDI, repertoire dissimilarity index; PCA, principal component analysis.
